# Supplementary material for: Allele segregation analysis of F1 hybrids between independent Brassica allohexaploid lineages
Source: Chromosoma. 2022 May 5;131(3):147–61. doi: 10.1007/s00412-022-00774-3 (PMC9470611; doi:10.1007/s00412-022-00774-3)
Supplement: Supplementary file 1 — Supplementary file1 (PDF 2516 KB) [file 412_2022_774_MOESM1_ESM.pdf]

Allele segregation analysis of F_1 hybrids between independent *Brassica* allohexaploid lineages

Daniela Quezada-Martinez[1, 2], Jun Zou[3], Wenshan Zhang[3], Jinling Meng[3], Jacqueline Batley[4], Annaliese S. Mason[1, 2,*]

[1] Plant Breeding Department, University of Bonn, 53115 Bonn, Germany

[2] Plant Breeding Department, Justus Liebig University, 35392 Giessen, Germany

[3] National Key Laboratory of Crop Genetic Improvement, Huazhong Agricultural University, Wuhan, China

[4] School of Biological Sciences, The University of Western Australia, Crawley 6009, Australia

[*] corresponding author: annaliese.mason@uni-bonn.de

ORCID: 0000-0003-0470-9925 (D.Q.M.), 0000-0002-5391-5824 (J.B.), 0000-0003-2701-7964 (A.S.M.)

Supplementary Information

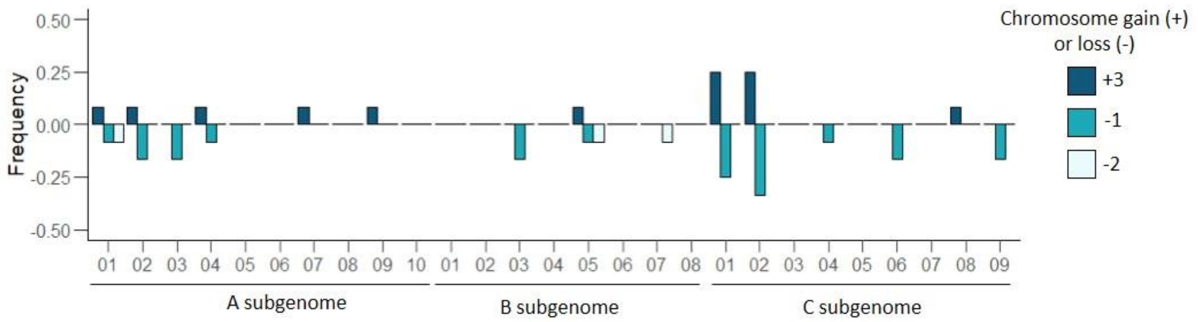

Supplementary Fig. 1 Frequency of chromosome variation in allohexaploid carirapa (*Brassica carinata* × *Brassica rapa*) and NCJ (*Brassica napus* × *Brassica carinata* × *Brassica juncea*) allohexaploid types. Whole chromosome changes (gain or loss) in 12 different allohexaploid plants across the three *Brassica* genomes (A, B and C)

a.

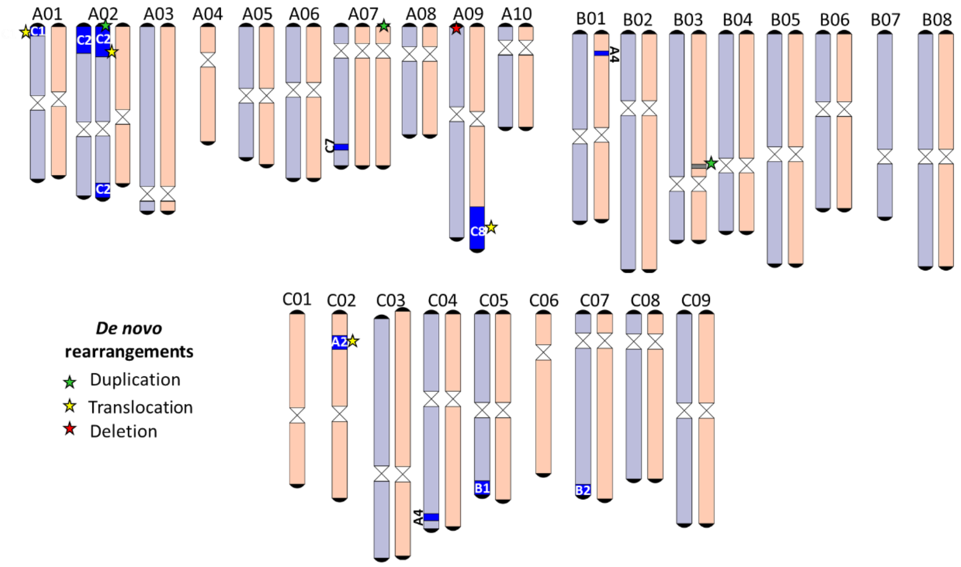

b.

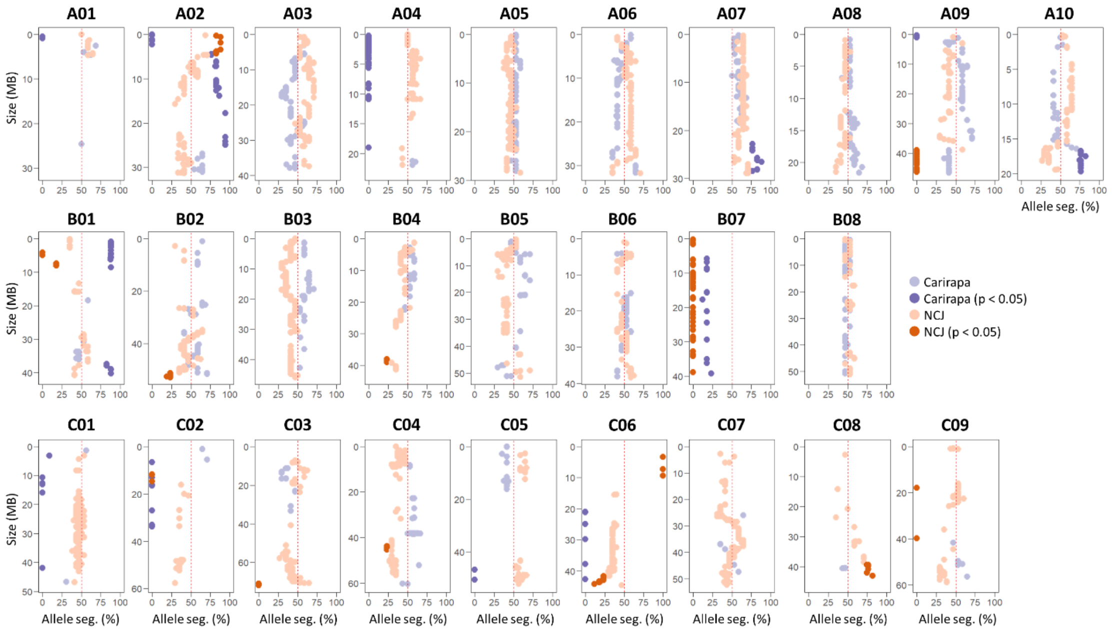

Supplementary Fig. 2 **a** Molecular karyotype and **b** allele segregation for F_1 hybrid population 4. Chromosomes are colored based on hexaploid parent: carirapa in purple, NCJ in orange. Rearrangements are colored in blue. Gray areas in the chromosome correspond to doubled regions with unknown locations in the genome. *De novo* translocations (present in the F_1 hybrid but not in the parents) are marked with a star in a different color depending on the type (see legend). Chromosome sizes are represented in megabases (Mb). Expected segregation ratio of the alleles (50%) is marked with a red dotted line for each chromosome. Significant allele distortion (X^2 test, $p < 0.05$) is indicated with dark orange (NCJ) or dark purple (carirapa). Seg. = segregation

a.

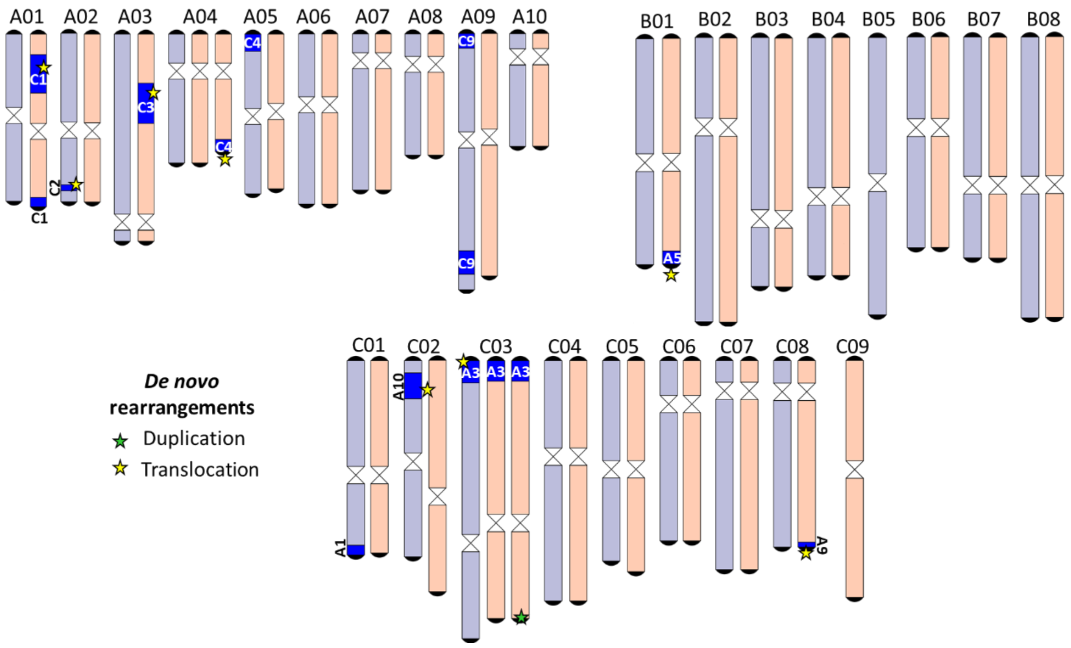

b.

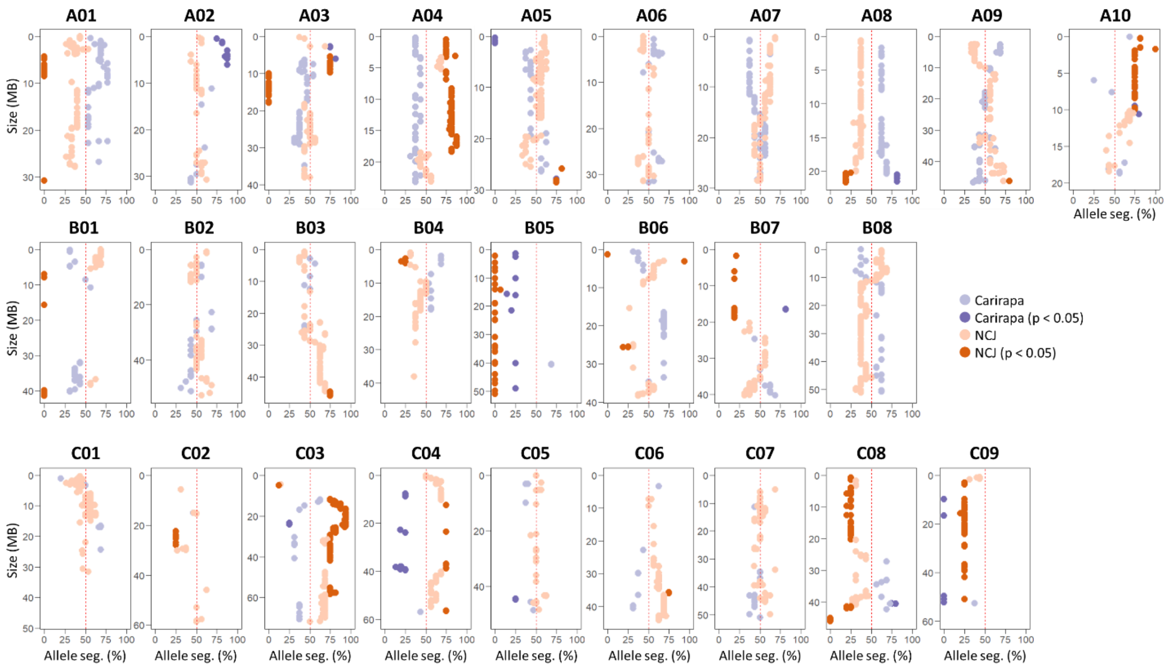

Supplementary Fig. 3 **a** Molecular karyotype and **b** allele segregation for F_1 hybrid population 5. Chromosomes are colored based on hexaploid parent: carirapa in purple, NCJ in orange. Rearrangements are colored in blue. *De novo* translocations (present in the F_1 hybrid but not in the parents) are marked with a star in a different color depending on the type (see legend). Chromosome sizes are represented in megabases (Mb). Expected segregation ratio of the alleles (50%) is marked with a red dotted line for each chromosome. Significant allele distortion (X^2 test, $p < 0.05$) is indicated with dark orange (NCJ) or dark purple (carirapa). Seg. = segregation

a.

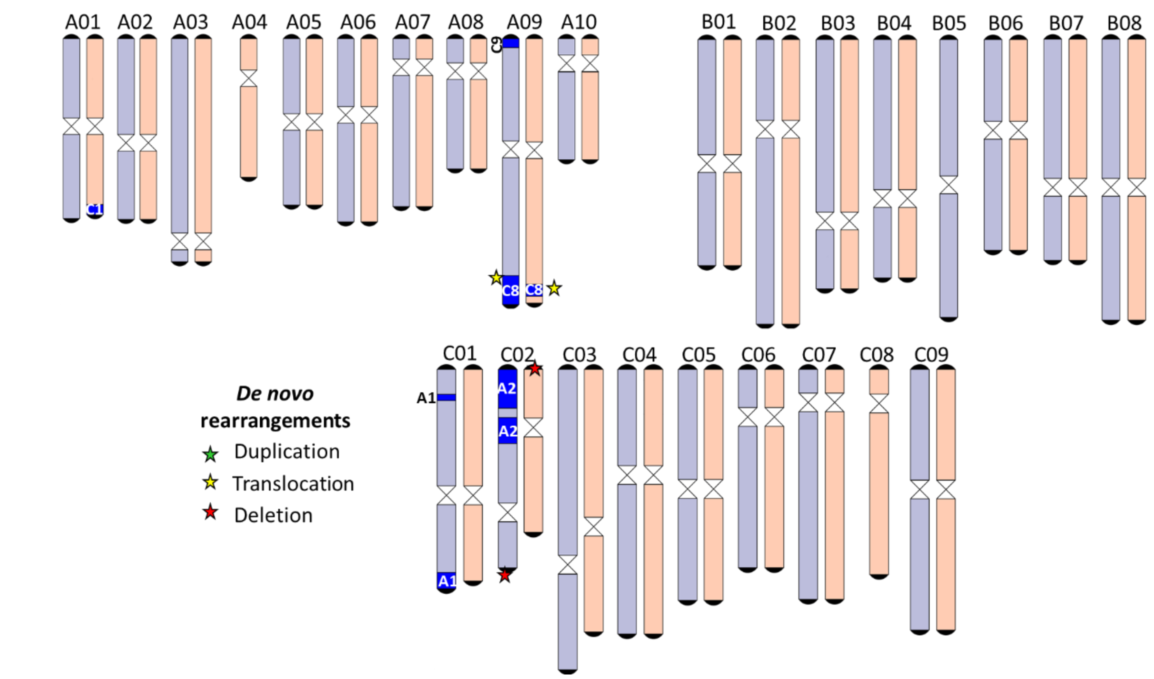

b.

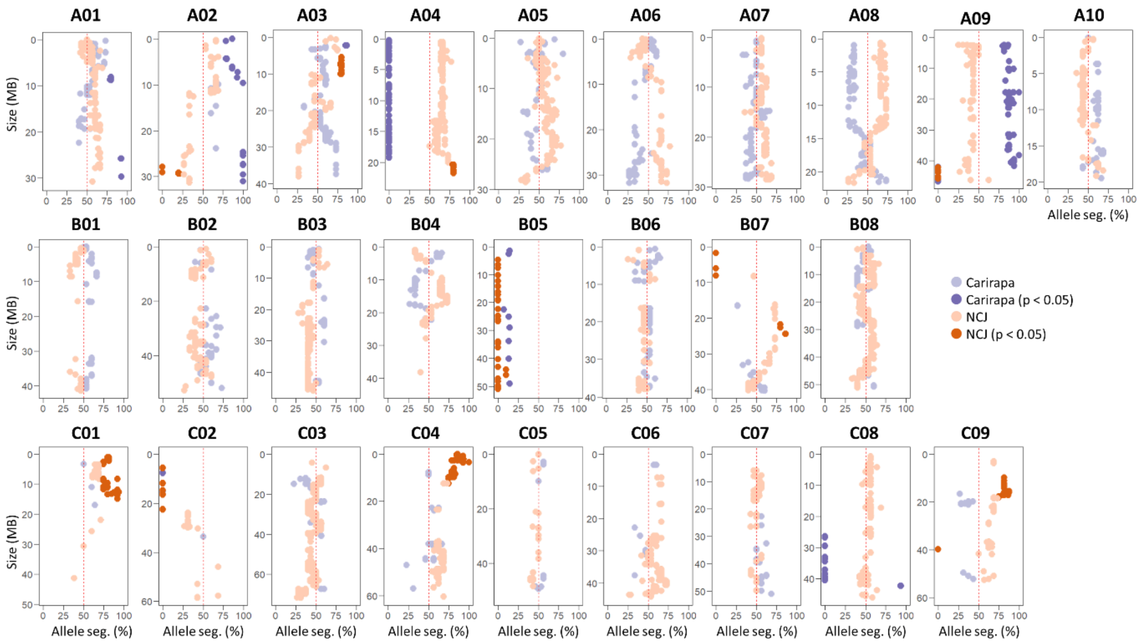

Supplementary Fig. 4 a Molecular karyotype and **b** allele segregation for F_1 hybrid population 6. Chromosomes are colored based on hexaploid parent: carirapa in purple, NCJ in orange. Rearrangements are colored in blue. *De novo* translocations (present in the F_1 hybrid but not in the parents) are marked with a star in a different color depending on the type (see legend). Chromosome sizes are represented in megabases (Mb). Expected segregation ratio of the alleles (50%) is marked with a red dotted line for each chromosome. Significant allele distortion (X^2 test, $p < 0.05$) is indicated with dark orange (NCJ) or dark purple (carirapa). Seg. = segregation

a.

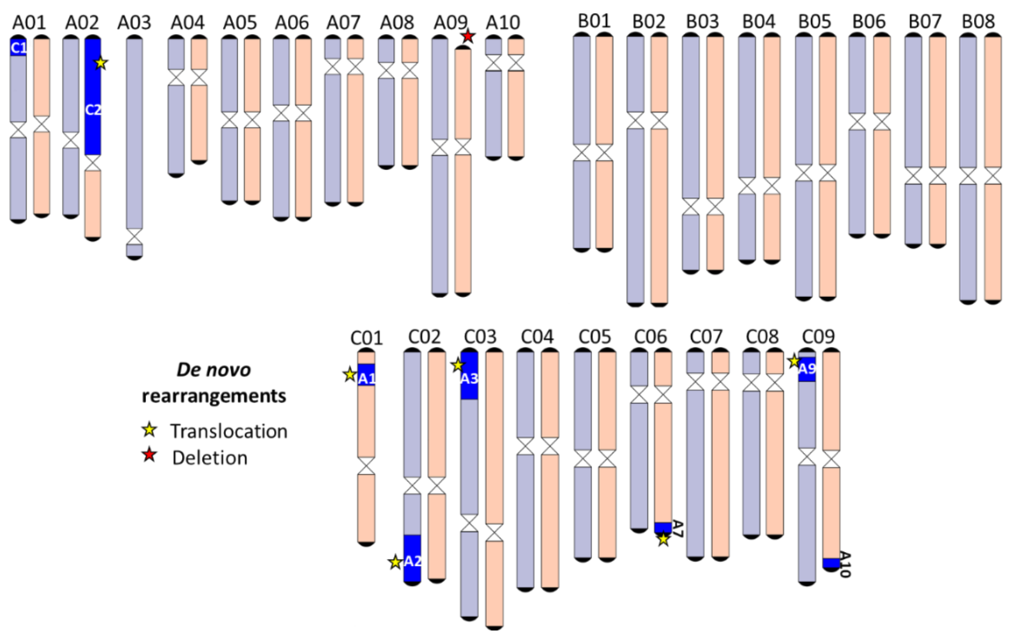

b.

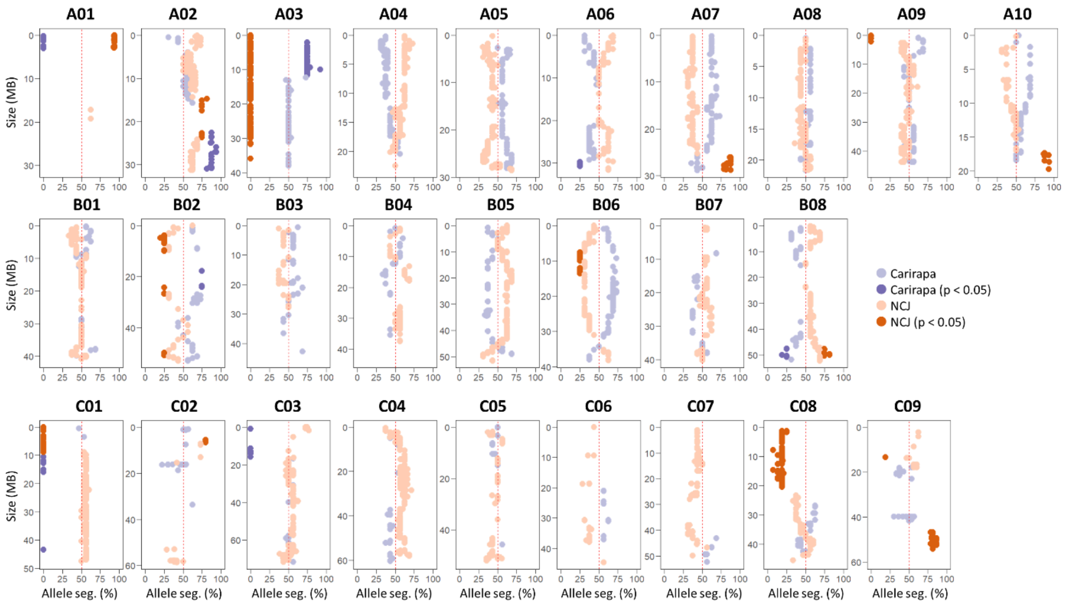

Supplementary Fig. 5 **a** Molecular karyotype and **b** allele segregation for F_1 hybrid population 8. Chromosomes are colored based on hexaploid parent: carirapa in purple, NCJ in orange. Rearrangements are colored in blue. Gray areas in the chromosome correspond to doubled regions with unknown locations in the genome. *De novo* translocations (present in the F_1 hybrid but not in the parents) are marked with a star in a different color depending on the type (see legend). Chromosome sizes are represented in megabases (Mb). Expected segregation ratio of the alleles (50%) is marked with a red dotted line for each chromosome. Significant allele distortion (X^2 test, $p < 0.05$) is indicated with dark orange (NCJ) or dark purple (carirapa). Seg. = segregation

Supplementary Data

Supplementary Table 1 Plant material used in the production of F1 hybrids between carirapa and NCJ hexaploids

Supplementary Table 2 Illumina Infinium *Brassica* 90K genotyping array data for the *Brassica* A-, B-, and C- genomes on the *Brassica napus* Darmor-bzh v8.1 and *B. nigra* Ni100 SR reference genomes for *Brassica* control species, *Brassica* hexaploid "NCJ", "Carirapa", F1 hybrids, test-cross parents, and test-cross populations. Allele AA, BB = homozygous, AB = heterozygous, and NC = not-called

Supplementary Table 3 Illumina Infinium *Brassica* 90K SNP array logR ratio data for the *Brassica* A-, B-, and C- genomes on the *Brassica napus* Darmor-*bzh* v8.1 and *B. nigra* Ni100 SR reference genomes for *Brassica* control species, *Brassica* hexaploid "NCJ", "Carirapa", F1 hybrids, test-cross parents, and test-cross populations

Supplementary Table 4 Cleaned Illumina Infinium Brassica genotyping array and logR ratio data for the Brassica A-, B-, and C- genomes on the B. napus Darmor-bzh v8.1 and B. nigra reference genomes for Brassica control species, Brassica hexaploid "NCJ", "Carirapa", F_1 hybrid, test-cross parent, and test-cross population for the five analyzed populations. Centromere location interval per chromosome is colored in purple. Alleles are colores as: no call (NC) = gray, AA = yellow, AB = light-green, BB = green. Log2 R ratios are colored as: <-2 = red (missing both copies), between -0.2 and -0.5 = orange (missing one copy), between 0.2 and 0.5 = light-blue (extra copy), >0.5 = blue (extra copy +3).
